# Supplementary figures and images for: An in vitro carcinogenesis model for cervical cancer harboring episomal form of HPV16
Source: PLoS One. 2023 Feb 10;18(2):e0281069. doi: 10.1371/journal.pone.0281069 (PMC9916646; doi:10.1371/journal.pone.0281069)

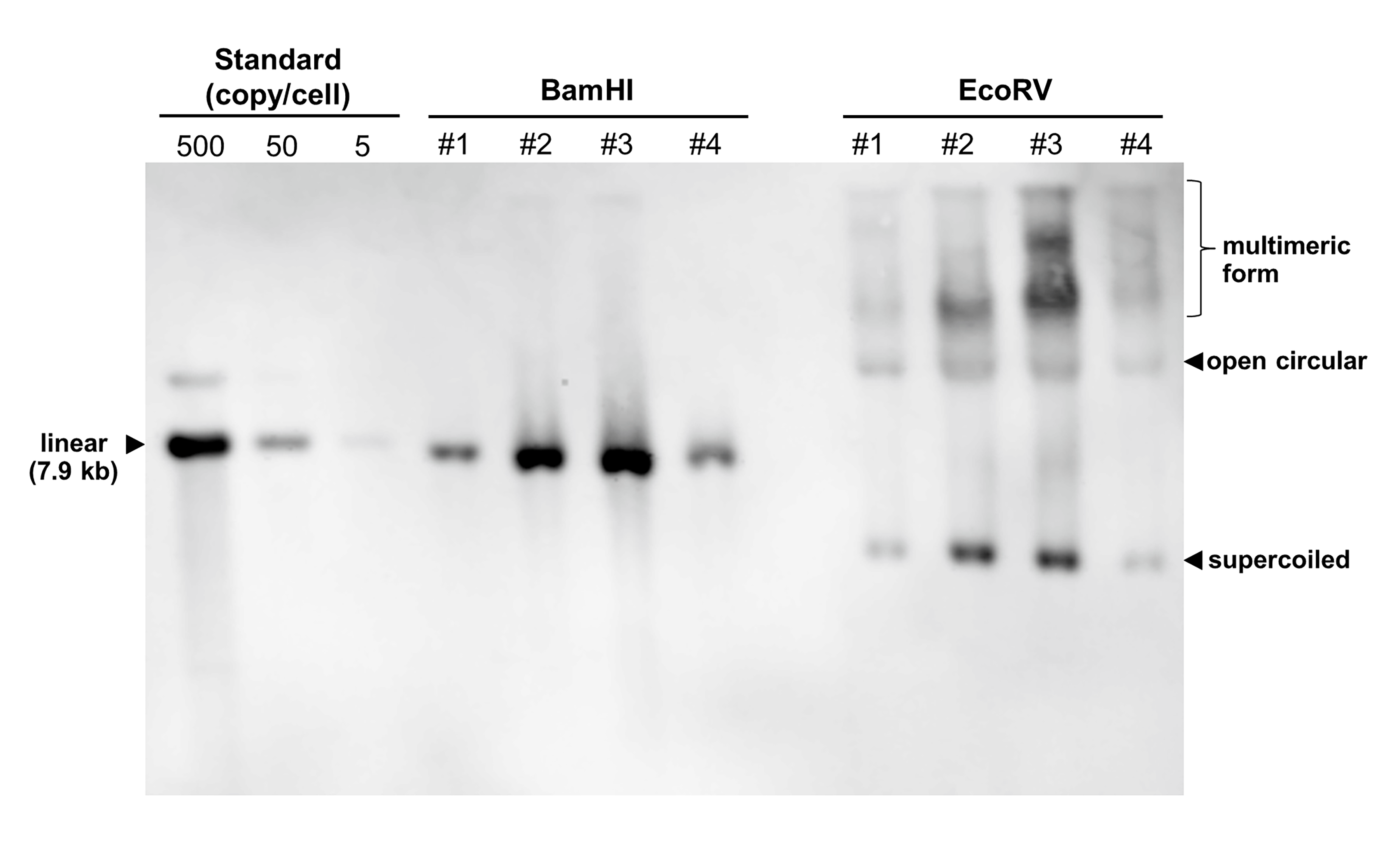

Supplement: S1 Fig — BamHI or EcoRV-digested total DNA isolated from HCK1T/16epi after transfection with PB-TAC-ERN-MYC-F2A1-3xFLAG-PIK3CAE545K and CSII-TRE-Tight-HA-MEK1DD plasmid are shown. Digestion with BamHI, which cuts the HPV16 genome once, produced results of the expected size for the HPV16 genome. Digestion with EcoRV, which does not cut the HPV16 genome, showed open circular and supercoiled plasmid of HPV16 genome. The BamHI-linearized HPV16 plasmid was used for length and copy number standards. #1: HCK1T/HPV16epi (p35); #2: HCK1T/HPV16epi/MYC-PIK3CAE454K/MEK1DD (p63); #3: HCK1T/HPV16epi/MYC-PIK3CAE454K/MEK1DD (p47); #4: HCK1T-HPV16epi/MYC-PIK3CAE454K (p67). (TIF) [file pone.0281069.s001.tif]

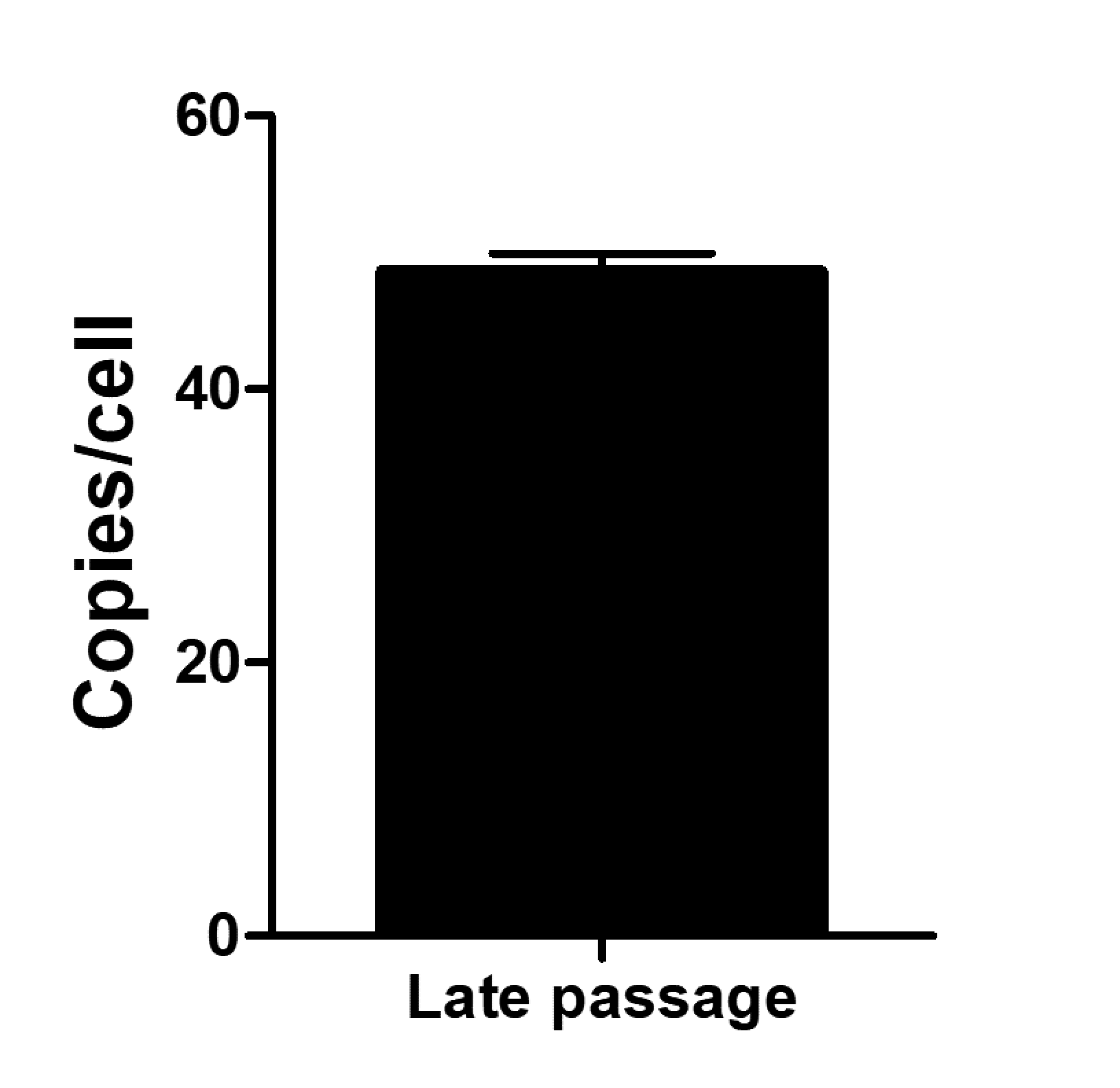

Supplement: S2 Fig — The bar represents the mean of triplicate values ± SEM. (TIF) [file pone.0281069.s002.tif]

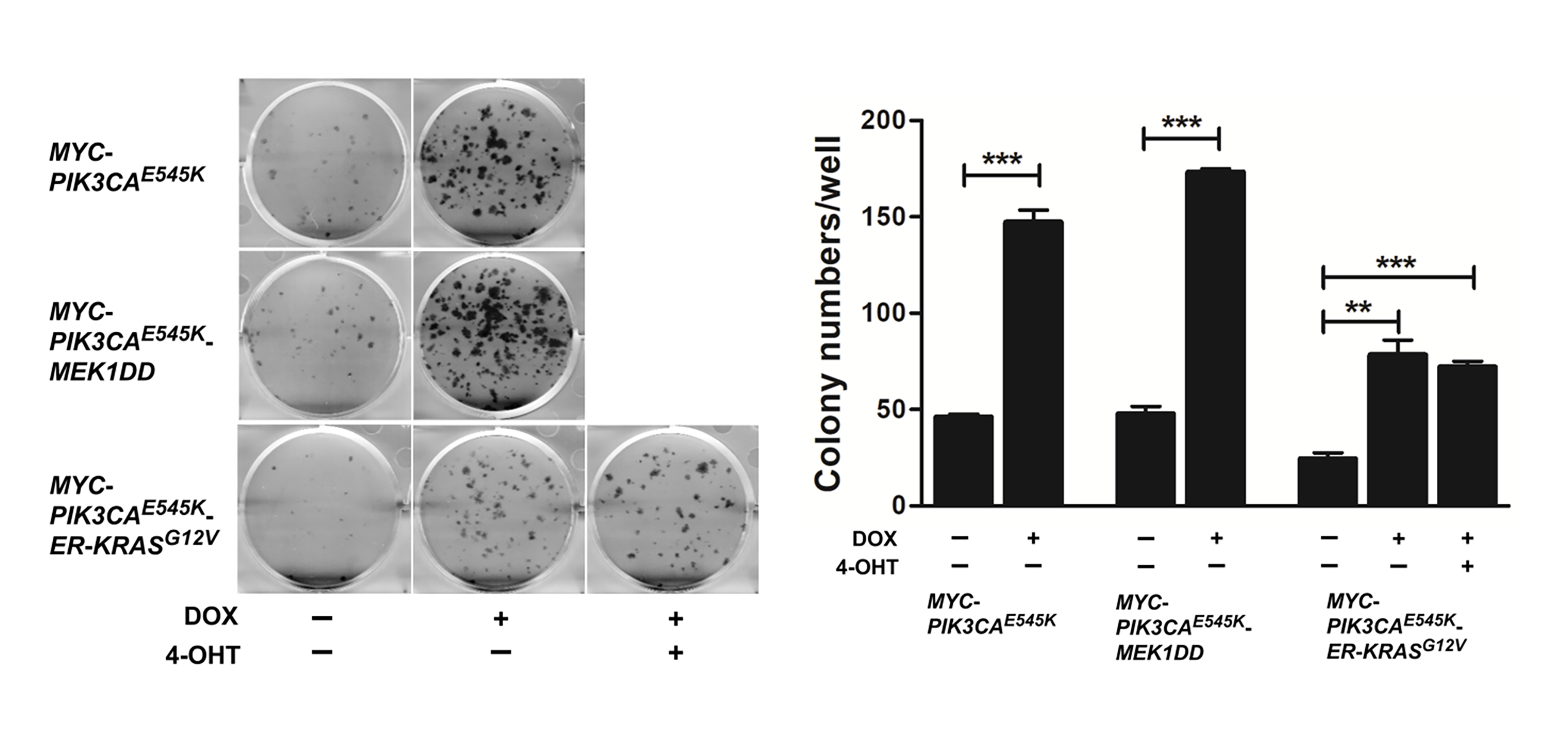

Supplement: S3 Fig — Each bar represents the mean of triplicate values ± SEM. **P ≤ 0.01, ***P ≤ 0.001. (TIF) [file pone.0281069.s003.tif]

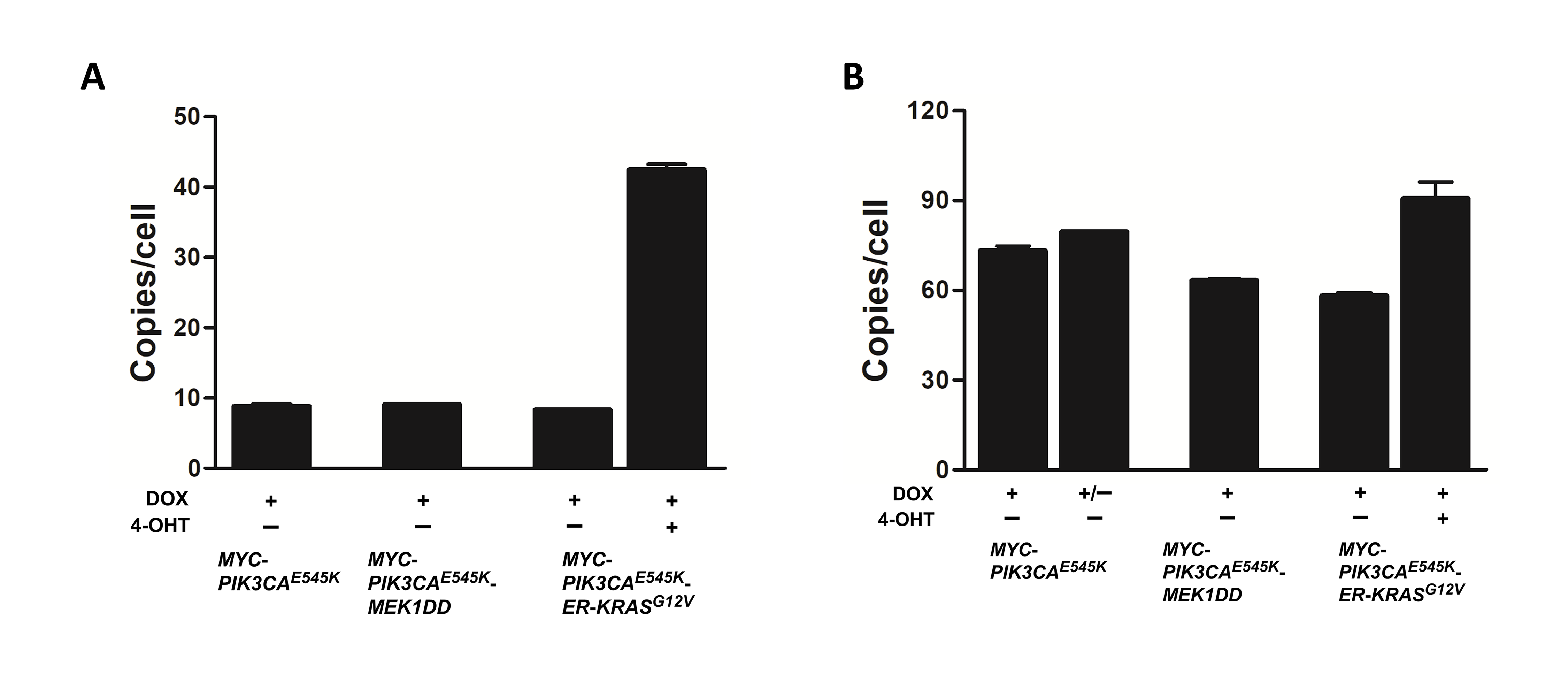

Supplement: S4 Fig — The copy number of HPV16 genomes in tumor tissues of early-passage (A) and late-passage (B) cells were determined by qPCR using primers specific to the E6 and L2 ORFs of HPV16. Each bar represents the mean of triplicate values ± SEM. (TIF) [file pone.0281069.s004.tif]
