# Supplementary material for: An in vitro carcinogenesis model for cervical cancer harboring episomal form of HPV16
Source: PLoS One. 2023 Feb 10;18(2):e0281069. doi: 10.1371/journal.pone.0281069 (PMC9916646; doi:10.1371/journal.pone.0281069)
Supplement: S1 Table — (DOCX) [file pone.0281069.s005.docx]

**Supporting information**

| **Cells** | **Passages** | **DOX** | **4-OHT** | **Number of tumors per sites of injection (weeks)** |
| --- | --- | --- | --- | --- |
| **Early passage** |  |  |  |  |
| HCK1T/16epi/*MYC-PIK3CA^E545K^* | (p45) | - | - | 0/4 |
| HCK1T/16epi/*MYC-PIK3CA^E545K^* | (p45) | + | - | 0/4 |
| HCK1T/16epi/*MYC-PIK3CA^E545K^* | (p45) | + | - | 4/4 (8) |
| HCK1T/16epi/*MYC-PIK3CA^E545K^-MEK1DD* | (p45) | + | - | 4/4 (3) |
| HCK1T/16epi/*MYC-PIK3CA^E545K^-ER-KRAS^G12V^* | (p45) | + | - | 4/4 (8) |
| HCK1T/16epi/*MYC-PIK3CA^E545K^-ER-KRAS^G12V^* | (p45) | + | + | 4/4 (4) |
| **Late passage** |  |  |  |  |
| HCK1T/16epi/*MYC-PIK3CA^E545K^* | (p61) | - | - | 0/4 |
| HCK1T/16epi/*MYC-PIK3CA^E545K^* | (p61) | + | - | 4/4 (5) |
| HCK1T/16epi/*MYC-PIK3CA^E545K^* | (p61) | +/- | - | 4/4 (5) |
| HCK1T/16epi/*MYC-PIK3CA^E545K^-MEK1DD* | (p61) | + | - | 4/4 (2) |
| HCK1T/16epi/*MYC-PIK3CA^E545K^-ER-KRAS^G12V^* | (p61) | + | - | 4/4 (5) |
| HCK1T/16epi/*MYC-PIK3CA^E545K^-ER-KRAS^G12V^* | (p61) | + | + | 4/4 (3) |
| NOTE: (p); the passage number of the cells used for subcutaneous injection into mice. +; drug-treated condition, -; drug-untreated condition and +/-; drug was added and removed later. (); latency was determined as the time (weeks) taken before a palpable mass could be detected. | | | | |

**S1 Table.** Numbers of nude mice developing tumors after injection of 1x10^6^ HCK1T/16epi cells (per site) expressing *MYC*, *PIK3CA^E545K^*, *MEK1DD* and *ER-KRAS^G12V^*.
