# Supplementary material for: An in vitro carcinogenesis model for cervical cancer harboring episomal form of HPV16
Source: PLoS One. 2023 Feb 10;18(2):e0281069. doi: 10.1371/journal.pone.0281069 (PMC9916646; doi:10.1371/journal.pone.0281069)
Supplement: S1 Raw images — (PDF) [file pone.0281069.s006.pdf]

The original blot images

Western blotting procedure

Whole-cell proteins were extracted in a lysis buffer (50 mM Tris-HCl, 250 mM NaCl, 5 mM EDTA, 1% NP-40, 20% glycerol, 0.1% SDS, 1% Deoxycholate) supplemented with 5% (v/v) protease inhibitor cocktail (Nacalai Tesque, Kyoto, Japan) and phosphatase inhibitors (500 μM sodium orthovanadate, 100 mM sodium fluoride, 10 mM sodium pyrophosphate). Protein concentrations were determined by using the DC protein assay (Bio-Rad). The same amounts of the proteins (20 μg/lane) were electrophoresed on SDS-polyacrylamide gels and transferred to the Immobilon-P PVDF membrane (Millipore, Billerica, MA, USA). The membrane was cut into thin strips depending on the size of the protein interested and blocked with 5% skim milk in Tris-buffered saline with 0.1% Tween 20 (TBS-T) for 1 hour at room temperature then overnight at 4°C, then incubated with specific primary antibody for 2 hours at room temperature. After extensive washing with TBS-T, the membranes were incubated with a secondary antibody for 1 hour at room temperature and washed with TBS-T again. The LAS3000 charge-coupled-device imaging system (Fujifilm Co. Ltd, Tokyo, Japan) was employed for the detection of proteins visualized by Lumi-light plus western blotting substrate (Roche Applied Science, Penzberg, Germany).

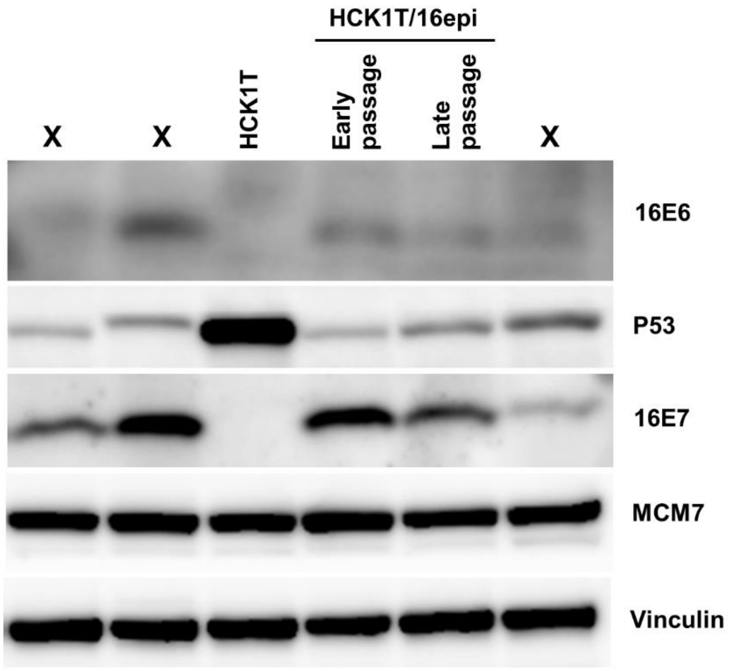

Fig 1E. Expression of HPV16 E6 and E7 oncoproteins was detected by western blotting using specific anti-HPV16 E6 and E7 antibodies and the levels of E6 and E7 were compared between early- and late-passage HCK1T/16epi cells. Parental HCK1T cells were included as a negative control. Levels of p53 and MCM7 proteins were also determined to show that E6 and E7 from episomal HPV16 genomes are able to reduce p53 or increase MCM7, respectively. Vinculin was included as a loading control.

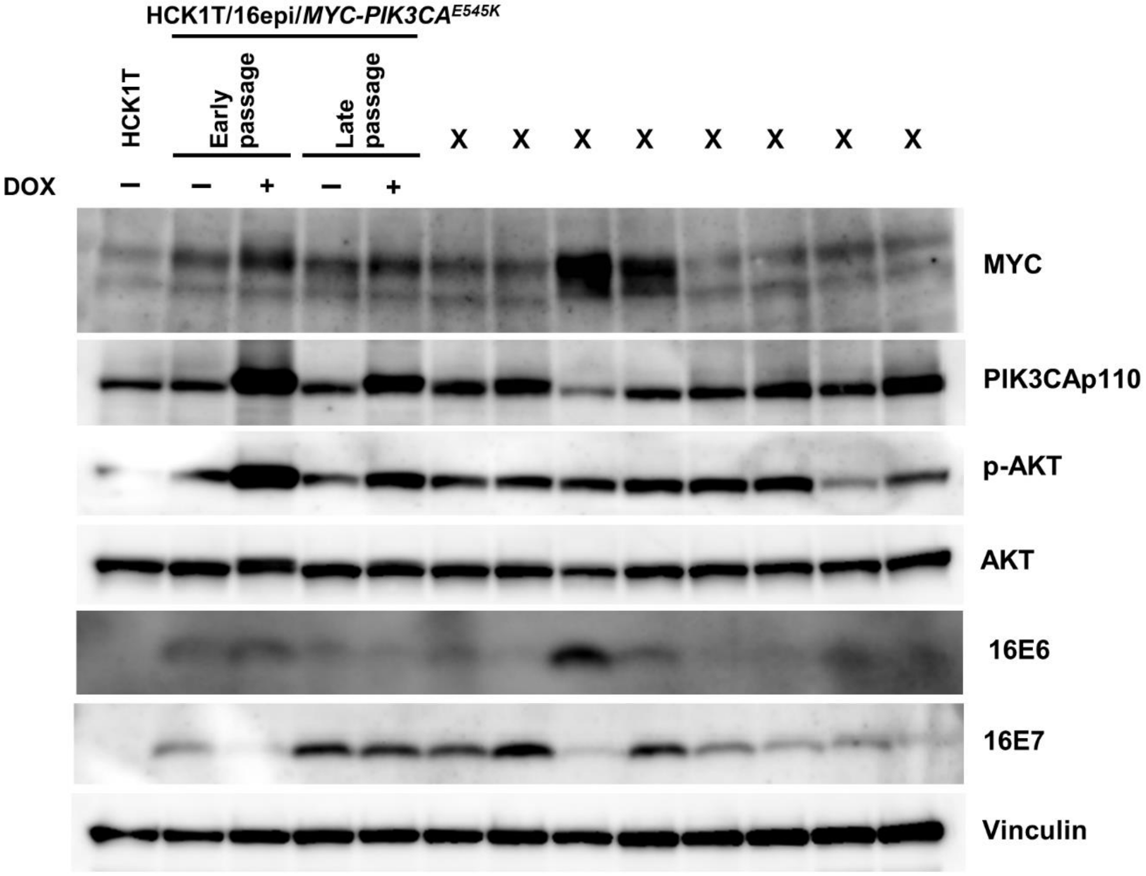

Fig 2B. The expression of individual transgenes including MYC and PIK3CAp110 and their downstream target proteins including AKT and p-AKT were detected by western blotting. The expression levels of HPV16 E6 and E7 proteins in HCK1T/16epi cells were also examined. Vinculin was included as a loading control.

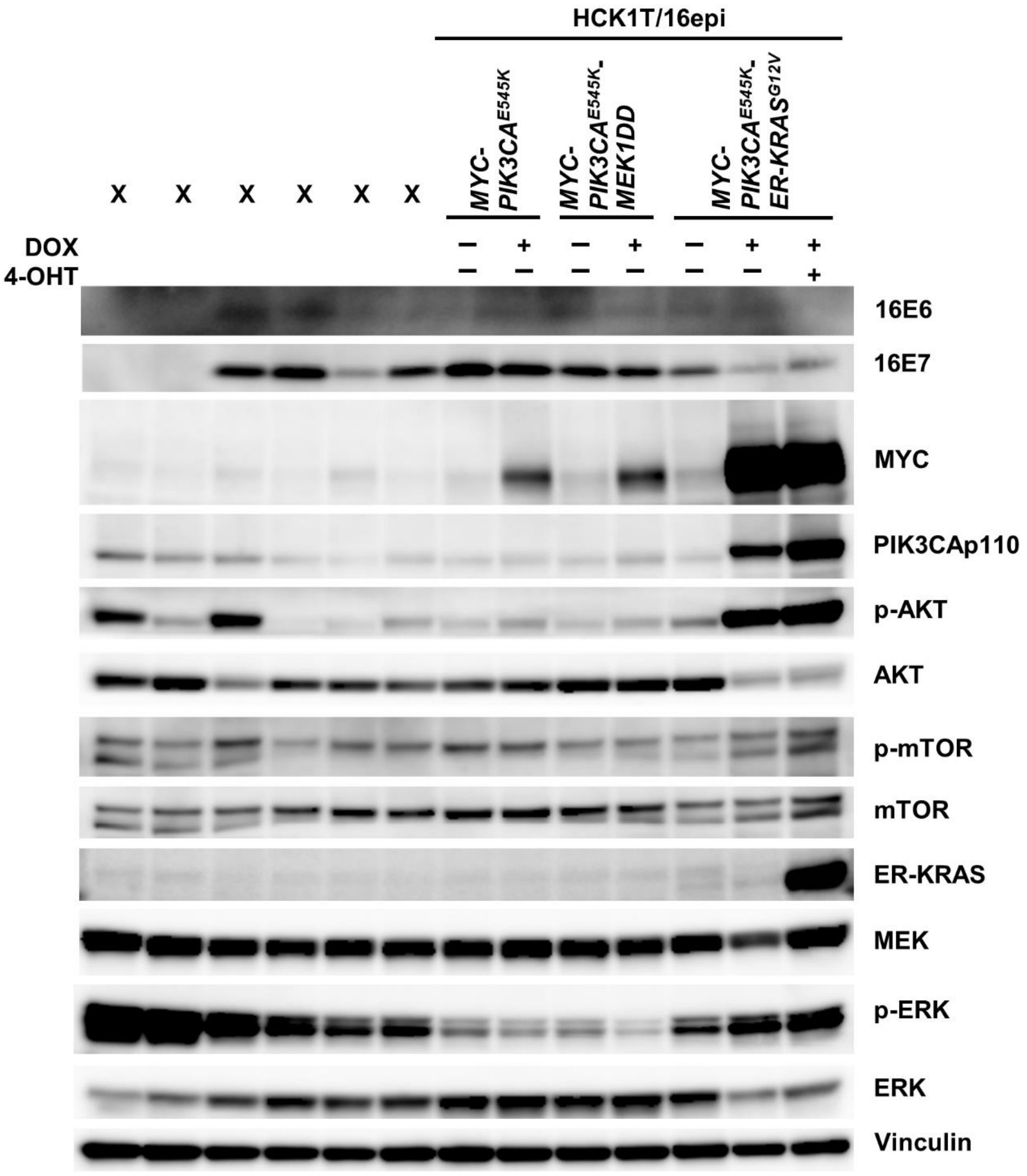

Fig 3E. The expression of individual transgenes including MYC, PIK3CAp110, MEK and KRAS, and their downstream target proteins including AKT; p-AKT, mTOR; p-mTOR and ERK; p-ERK were detected by western blotting, in the cells from early passage.

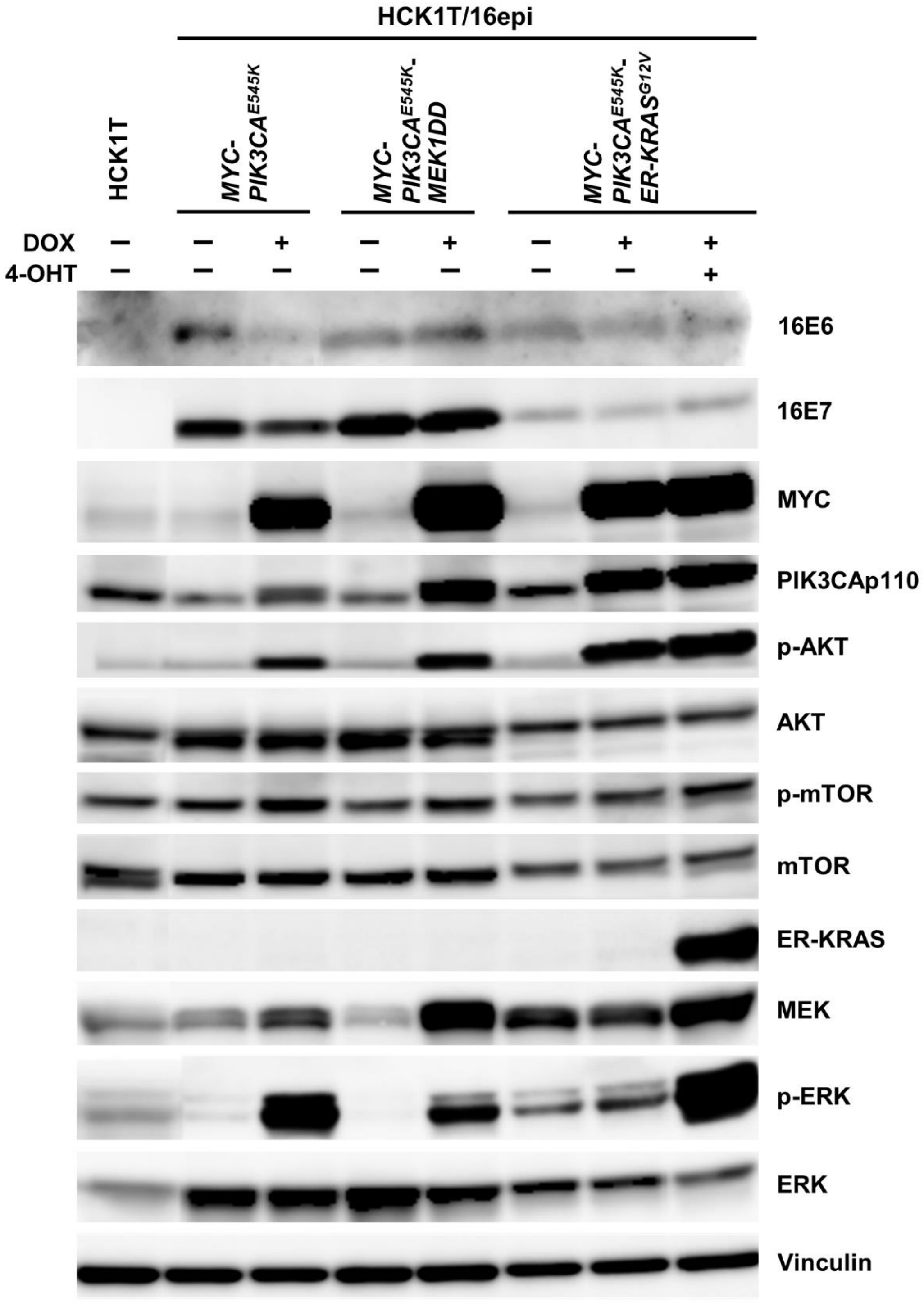

Fig 3F. The expression of individual transgenes including MYC, PIK3CAp110, MEK and KRAS, and their downstream target proteins including AKT; p-AKT, mTOR; p-mTOR and ERK; p-ERK were detected by western blotting, in the cells from late passage.
