# Supplementary material for: An in vitro carcinogenesis model for cervical cancer harboring episomal form of HPV16
Source: PLoS One. 2023 Feb 10;18(2):e0281069. doi: 10.1371/journal.pone.0281069 (PMC9916646; doi:10.1371/journal.pone.0281069)
Supplement: Attachment — Submitted filename: Response to Reviewers.docx [file pone.0281069.s008.docx]

**Response to Reviewers**

PONE-D-22-22148R1

An in vitro carcinogenesis model for cervical cancer harboring episomal form of HPV16

PLOS ONE

**Review Comments to the Author**

Please use the space provided to explain your answers to the questions above. You may also include additional comments for the author, including concerns about dual publication, research ethics, or publication ethics. (Please upload your review as an attachment if it exceeds 20,000 characters)

**Response to the Reviewer #1:**

**Reviewer #1:** The authors replied to all points raised, but did not perform a single additional experiment.

- Most importantly, their answers confirmed that, indeed, not groups of mice were used, but a single mouse per condition for figures 2 and 4! The error bars just stem from the fact that 4 tumors were set per mouse.
- It is completely unacceptable to base any conclusions on experiments with a single animal per condition. It is now clear why there are such large differences between the exact same conditions between Figure 2 and Figure 4, or within Figure 4. As these are not groups of mice, but single animals, differences are to be expected. For example because single mice can have different drinking behaviours, and thus take in less DOX or 4-OHT. This is exactly the reason why there have to be several mice in a group.
- A proper study should involve a biostatistician before the experiment is conducted, an estimation of the size of the effect to be tested (and the required statistical significance), and then a calculation of the necessary number of mice per experimental group.

**Reply:** Firstly, we would like to thank you for the comment, and regarding the number of mice that we used in this experiment; we would like to explain that our research plan to minimize the use of mice in order to comply with the research and achieve the objectives of the study. However, we are aware of important biological effects that can be lost if too few mice are used. We also looked at previous studies on the number of mice used in experiments similar to this study. It was found that one mouse per condition was used and four or six sites per mouse were also cells injected (Narisawa-Saito et. al., 2008, Narisawa-Saito et. al., 2012).

For the advantage of using one mouse per condition is that it minimizes the genetic variation and environmental effects that can be occurred if multiple mice are used. But the advantage of using multiple mice per condition, as you suggest, can reduce the impact that might have on experiments involving different animal behavior, such as different drinking habits.

However, even if there are factors related to animal behavior outside of the control as mentioned and affecting the experiment. In this experiment, we used mice as a suitable study and have followed the guidelines for the proper use of animals in scientific work.

We sincerely apologize for not being able to repeat the experiment or do more experiments as you suggest. Because our research group has changed its approach of studying and some members have changed jobs to a new organization. However, the research presented here is properly confirmed by our research group and considered to be suitable for publication and dissemination.

References

Narisawa-Saito M, Yoshimatsu Y, Ohno S-i, Yugawa T, Egawa N, Fujita M, et al. An in vitro multistep carcinogenesis model for human cervical cancer. Cancer research. 2008;68(14):5699-705.

Narisawa-Saito M, Inagawa Y, Yoshimatsu Y, Haga K, Tanaka K, Egawa N, et al. A critical role of MYC for transformation of human cells by HPV16 E6E7 and oncogenic HRAS. Carcinogenesis. 2012;33(4):910-7.

- The authors also did not get my point that groups (or single mice) that did not receive the transgene-inducing agent should behave like the groups without the transgene. They continue just to describe the findings that fit their hypothesis, and not the findings (shown in figures) that do not. E.g. in panels 3E and 3F.

**Reply:** We thank the reviewer for the comment.

First of all, we would like to apologize for the previous answer that did not understand the point you wish to discuss. However, we agree with you on the point that mice without transgene inducers should have the same properties as mice without transgene. As shown in Figure 4, mice that were not received transgene inducers did not develop tumors. In addition, mice given the transgene inducer develop tumors early. But when the doping was stopped, the tumor size gradually decreased.

In figures 3E and 3F, protein expression was examined by western blot in HCK1T/16epi cells treated with transgene inducers to confirm the function of those transgenes in comparison with HCK1T parental cells. As you can see in Figures 3E and 3F, increased phosphorylation of AKT and mTOR which are the downstream target of PIK3CA, and also ERK which is the downstream target of KRAS and MEK confirmed that induced transgenes are functionally active.

However, from Figure 3F, it can be seen that in HCK1T/16epi with MYC-PIK3CA^E545K^-ER-KRAS^G12V^ cells that did not expose to transgene inducers had slightly higher expression levels of MEK and ERK proteins when compared with HCK1T parental cells. This result might be the effect from the leakage expression of transgenes that are commonly occurred in the constructed vectors and may affect on downstream target protein activation.

- I reiterate that the E6 blot in Figure 2B is not fit for publication, and that there is a loading problem in Figures 3E (last two lanes) and 3F (last three lanes), which can also be seen in the Vinculin staining. It is not sufficient just to state that there is no problem, the experiments should be repeated until proper blots can be shown.

**Reply:** We thank the reviewer for the comment and suggestion.

First of all, we would like to apologize for not being able to repeat the experiments as you suggest and sincerely apologize for the previous comment regarding the problem to detect E6 protein expression by western blot. According to vinculin staining, we found that the E6 protein expression was detected at low levels when compared with E6 gene expression level in Figure1C and the protein band is unclear. This problem might be effected from protein loading and the antibody clone used. However, we try to make it more clear as shown in Figure 2B and E6 gene expression can be confirmed by the level of mRNA detected in the cells, shown in Figure 1C.

We already added the description in the result section of the revised manuscript with track changes. Please see the result section in line 319-322.

- Regarding the suggested experiment, to test if cells that contain activated MYC and RAS – described as sufficient to transform rodent cells in the discussion – still need HPV oncogenes at all, the authors just wrote “We strongly agree with what you suggest”. As they agree, they really need to perform this experiment!

**Reply:** We thank the reviewer for the comment and suggestion. We think that your hypothesis is an interesting point and it is necessary to explore further with many experiments. However, we sincerely apologize for not being able to do more experiments as you suggest. As we already tell you that our research group has changed its approach of studying and some members have changed jobs to a new organization. As a result, we were unable to perform this experiment.

**Response to the Reviewer #3:**

**Reviewer #3:** The authors in this manuscript described the full transformation of HCK1T cell line, carrying episomal HPV16 genomes, following ectopic expression of MYC and PIK3CAE545K. Overall the manuscript is well written and study results well presented

**Reply:** We thank the reviewer for the comment.
